# Ill Literates or Illiterates? Investigating the eHealth Literacy of Users of Online Health Communities

**DOI:** 10.2196/jmir.7372

**Published:** 2017-10-04

**Authors:** Gregor Petrič, Sara Atanasova, Tanja Kamin

**Affiliations:** ^1^ Centre for Methodology and Informatics Faculty of Social Sciences University of Ljubljana Ljubljana Slovenia; ^2^ Centre for Social Psychology Faculty of Social Sciences University of Ljubljana Ljubljana Slovenia

**Keywords:** health literacy, online health community, user types, weights and measures, survey methods

## Abstract

**Background:**

Electronic health (eHealth) literacy is an important skill that allows patients to navigate intelligibly through the vast, often misleading Web-based world. Although eHealth literacy has been investigated in general and specific demographic populations, it has not yet been analyzed on users of online health communities (OHCs). Evidence shows that OHCs are important Web 2.0 applications for patients for managing their health, but at the same time, warnings have been expressed regarding the quality and relevance of shared information. No studies exist that investigate levels of eHealth literacy among users of OHCs and differences in eHealth literacy between different types of users.

**Objective:**

The study aimed to investigate eHealth literacy across different types of users of OHCs based on a revised and extended eHealth literacy scale (eHEALS).

**Methods:**

The study was based on a cross-sectional Web survey on a simple random sample of 15,000 registered users of the most popular general OHC in Slovenia. The final sample comprised 644 users of the studied OHC. An extended eHEALS (eHEALS-E) was tested with factor analytical procedures, whereas user types were identified with a hierarchical clustering algorithm. The research question was analyzed with analysis of variance (ANOVA) procedure and pairwise comparison tests.

**Results:**

Factor analysis of the revised and extended eHEALS revealed six dimensions: awareness of sources, recognizing quality and meaning, understanding information, perceived efficiency, validating information, and being smart on the Net. The factor solution demonstrates a good fit to the data (root mean square error of approximation [RMSEA]=.059). The most developed dimension of eHEALS-E is awareness of different Internet sources (mean=3.98, standard deviation [SD]=0.61), whereas the least developed is understanding information (mean=3.11, SD=0.75). Clustering resulted in four user types: active help-seekers (48.3%, 311/644), lurkers (31.8%, 205/644), core relational users (16.9%, 109/644), and low-engaged users (3%, 19/644). Analysis of the research question showed statistically significant differences among user types across all six dimensions of eHEALS-E. Most notably, core relational users performed worse than lurkers on the validating information dimension (*P*=.01) and worse than active help-seekers on the being smart on the Net dimension (*P*=.05). Active help-seekers have the highest scores in all dimensions of the eHEALS-E, whereas low-engaged users have statistically significantly lower scores on all dimensions of the eHEALS-E in comparison with the other groups.

**Conclusions:**

Those who are looking for advice and support in OHCs by making queries are well equipped with eHealth literacy skills to filter potential misinformation and detect bad advice. However, core relational users (who produce the most content in OHCs) have less-developed skills for cross-validating the information obtained and navigating successfully through the perils of the online world. Site managers should monitor their activity to avoid the spread of misinformation that might lead to unhealthy practices.

## Introduction

### eHealth Literacy and Online Health Communities

With the undeniable prevalence of self-managing patients who are building expertise and making health decisions based on experiences in the online world, the recent upsurge of research on electronic health (eHealth) literacy is not surprising [1-6]. eHealth literacy originates from the multidimensional, dynamic concept of health literacy [7], which pertains to the cognitive and social skills for obtaining, processing, understanding, communicating, and using health-related information to function in the contemporary health care environment and to engage in appropriate self-care [7-10]. In addition to the dimension of health literacy, eHealth literacy embraces the human ability to meaningfully and efficiently navigate vast online spaces and is vital for the contemporary Internet patient to be able to make informed decisions that lead to successful health self-management, more effective contact with physicians, and positive health outcomes [2,6,11-13]. This literacy has been investigated mostly on general or specific demographic segments of populations in different national contexts, and it has not yet emerged as a topic researched among users of online health communities (OHCs). We believe that this is a critical gap that research needs to address for at least two reasons.

First, OHCs as a specific subset of online communities are commonly used Web-based applications, integrating discussion board communicative spaces that are dedicated but not limited to health-related issues, where users (patients, caregivers, or other individuals interested in health-related issues) participate, interact with other users and health professional moderators (usually health care providers), or just observe others’ interactions. OHCs can range from small-knit groups dedicated to specific health conditions or they may encompass hundreds of thousands of users, covering a wide variety of health conditions, from general and acute issues to specific (chronic) conditions such as heart disease, diabetes, cancer, mental health issues, and so on [14]. In OHCs, users can obtain information before or after visiting a physician [15]; can receive social support, advice, and hints for coping with a health issue from other users; can face various perspectives of the health issue; and receive health consultations and clinical expertise from health professional moderators [16-20]. Moreover, OHCs are often hailed for the availability of rich information and psychosocial resources that enable patients and users in general to achieve psychological empowerment [16,19-23]. However, warnings and concerns [24-27] have been issued, backed by research evidence [28-30], indicating that OHCs can be places where users can be misguided and exposed to information of low relevance and questionable validity. Although OHCs are implementing mechanisms to minimize risks regarding misinformation by integrating health professional moderators [31-33] or artificial intelligence tools for filtering information [34-36], they are not immune to deceptions and exaggerations, which are characteristic of online phenomena in general [37]. Experiences with OHCs and consequent benefits or damages for health outcomes are thus largely dependent on the degree to which a user’s eHealth literacy is developed [1,38]. Users with low eHealth literacy can fall a prey to advertising misguidance [39] and might also be unable to detect irrelevant or invalid information [40] or practices that can lead to very problematic health outcomes, for example, in the case of OHCs in which users have been stimulated to practice unhealthy lifestyles [41,42].

Second, another important aspect of eHealth literacy in OHCs is that they are typical Web 2.0 applications, where users are not only consumers of health-related information but also its producers. Users thus are involved not only in information-receiving communication processes but also in knowledge creation and sharing practices [36]. By posting messages in discussion threads, users in OHCs share experiences with health issues, offer advice and support to others, answer questions, chit-chat with other users, and share links to other websites [18,20,34,43]. In other words, by conducting such activities, users provide examples of health practices and educational material in general for other users [26].

### Types of OHC Users and Their eHealth Literacy

As participation in online environments demands certain levels of computer and media literacy, which are components of eHealth literacy [12,13], we might expect that those who create content (ie, posters) have higher eHealth literacy than those who do not create content (ie, lurkers). However, a recent study [44] showed that there is no correlation between eHealth literacy and participatory behavior in online environments. In other words, users who cocreate knowledge in OHCs are not necessarily more eHealth literate than lurkers. Current research does not provide insights into levels of eHealth literacy among posters. We believe this issue is immensely important, as posters’ eHealth literacy presents an important background against which knowledge in OHCs is produced.

In addressing the differences between those who consume and those who produce content in OHCs, we must consider recent reviews of the different types of participation in such online venues [27,34], which clearly show that the typologies of users in OHCs should go beyond the poster–lurker dichotomy [19,20]. Especially, the nomenclature for the types of posters varies greatly and is dependent on different metrics and approaches (see [34]). At a minimum, there exist at least three different, but not mutually exclusive, types of posters that are relevant while investigating eHealth literacy. One type, often called crisis-oriented users [45] or help-seekers [34], are users who typically produce query-based posts when searching for tailored answers to their specific needs. The second user type, which are often termed relational users [46], are more versatile, engaged in giving advice and support to others, and also involved in trivial conversation with other users, which is important for the sustainability of an online community [47]. The third type, commonly called superusers [48] or core users [49], are a small minority of those who create the majority of content and, most importantly, determine the tenor and the core knowledge base of the community.

As all these user types can have an important direct or indirect influence on other users [48,50], the question of their eHealth literacy in connection with their role in cocreating the social and informational terrain of OHCs is very relevant. Thus, the main aim of this paper was to investigate differences in eHealth literacy among various types of users of OHCs. In other words, the research question is—what is the level of eHealth literacy among various types of users of OHCs? First, we revised the common scale for measuring eHealth literacy (eHealth literacy scale or eHEALS) and proposed an extended version (eHEALS-E). Then empirical types of users were identified and compared regarding their degrees of eHealth literacy.

## Methods

### Procedure and Participants

The cross-sectional survey study was limited to users of Med.Over.Net (MON), the largest OHC in Slovenia that was established in 2000 and offers around 200 online discussion forums, of which the majority are moderated. In general, this OHC covers three types of online interactional spaces: (1) online counseling forums in which health professional moderators answer users’ queries; (2) social support group forums focused on specific symptoms or health conditions; and (3) general social forums dedicated to topics that are indirectly associated with health issues (parenting, food, relationships, etc). MON has, on average, more than 400,000 monthly visits and more than 70,000 registered users. This study was conducted in collaboration with the community managers of MON as part of their annual survey of user experiences and satisfaction with the OHC. The survey, in which respondents participated voluntarily and anonymously, was administered during June 2016 by the OHC provider, which followed ethical standards for administering scientific surveys. After clicking the link for the Web survey in the email, potential respondents were taken to an informed consent Web page with information about the purpose of the research and the length of the survey, an assurance that the data would be dealt with in accordance with national and European Union (EU) laws, information about the investigator, contact information, and a statement that the potential respondents were under no obligation to participate and that the aggregated results might be published. After giving their informed consent and clicking the Next button, respondents could start to fill out the survey. The survey was conducted on the platform english.1ka.si, open source online survey application that was developed at Centre for Social Informatics, Faculty of Social Sciences, University of Ljubljana. 1ka has mechanisms that disallow multiple entries by the same users. MON is a reputable Web service that treats all personal information (emails) in accordance with national and EU laws and protects data with standard security procedures, which include the deidentification of locally held data files, physical protection of hardware, and strong password protection. The authors of this study did not have access to the respondents’ emails and received an anonymized dataset that contained no identifiable personal information. Per the Code of Ethics for researchers at the University of Ljubljana [51], no institutional ethics approval was needed for this retrospective type of study. All research was conducted in line with the World Medical Association (WMA) Declaration of Helsinki on ethical principles for medical research involving human subjects.

The provider first designed a random sample of 40,000 registered users from the list of all registered users who visited MON at least once within the last 6 years. Approximately 15,000 of these registered users were randomly assigned to the Web survey used for this study, whereas approximately 25,000 users were randomly assigned to a second survey that mostly focused on users’ experiences with physicians and did not provide data for this study. Of approximately 15,000 potential respondents, 2147 clicked on a link on the Web survey, and 29.99% (644/2147) provided answers to items for the analysis of the research question. To present the sociodemographic characteristics of the sample, we performed missing values imputation on these variables, as they appeared at the end of the lengthy questionnaire and consequently contained a larger number of item nonresponse. More information about the Web survey can be found in the Checklist for Reporting Results of Internet E-Surveys (CHERRIES) in Multimedia Appendix 1.

The sample comprised 17.0% (109/644) men and 83.0% (535/644) women (Table 1). Respondents ranged in age from 15 to 90 years (mean=40.0, SD=10.3). More than half (67.7%, 436/644) of the respondents had at least a college degree, a large majority (77.1%, 497/644) were married or de facto married, 71.1% (458/644) were employed or self-employed, and 37.3% (240/644) claimed that they have a chronic or acute disease. The majority (58.5%, 377/644) of respondents use the OHC because of their own health issues, 23.0% (148/644) as caregivers, and 18.5% (119/644) for other purposes.

### Measures

#### Extended eHealth Literacy Scale (eHEALS-E)

The definition of eHealth literacy as “the ability to seek, find, understand, and appraise health information from electronic sources and apply the knowledge gained to addressing or solving a health problem” ([13]:1) underpins eHEALS, one of the most frequently used measurement instruments for eHealth literacy [2,6,52]. The eHEALS was originally developed for practical use in clinical settings [13] and thus comprises only 8 to 10 items. This number of items might be too small to grasp the complex essence of eHealth literacy, which integrates various literacies and comprises four components, which are as follows: accessing, understanding, appraising, and applying online information that is relevant to health [13]. In this light, it is unsurprising that studies show conflicting results regarding the unidimensionality of the scale [53] and that the scale, in general, lacks evidence of psychometric quality [6,54]. One author of the original scale [55] has called for improvement of the eHEALS. Therefore, we decided to revise the existing scale and offer an improved and extended version based on the original theoretical premises, addressing documented critical issues. In doing so, we followed a strict methodology for developing valid and reliable scales [56] and without any a priori limitations on a small number of items. In the initial item set, we retained all the items of the original eHEALS and introduced a small change by reverse coding two items with the intention of minimizing social desirability bias [57]. We developed an additional set of items by leaning on the essential elements of eHealth literacy as deduced from the definition of the concept [12]. These items thus pertain to the components of accessing, understanding, appraising, and applying relevant online health information, which are not well represented in the eHEALS and includes the following: knowing about or being aware of professional online resources, performing the search process, cross-validating health-related information obtained from the Internet, grasping meaning from the information obtained from the Internet, verifying the credibility of the online information, and maintaining a critical awareness of biases in Internet-based information. This last dimension is especially important in the context of recent warnings about filter bubbles [58,59] and the echo chamber effect [60], which point out that an individual user can unintentionally get locked in an information space that is seemingly open and objective but in reality is closed and biased, which in turn can have a problematic impact on health outcomes [49,61].

**Table 1:** Sample characteristics (N=644).

Variable		n (%)
**Gender**		
	Male	109 (17.0)
	Female	535 (83.0)
**Education**		
	Lower	47 (7.3)
	Middle	161 (25.0)
	Higher	436 (67.7)
**Labor market status**		
	School-age youth	25 (3.9)
	Worker, farmer	458 (71.1)
	Retired, unemployed, disabled	146 (22.7)
	Other	15 (2.3)
**Marital status**		
	Married or de facto married	497 (77.1)
	Single, divorced, widowed	147 (22.9)
**Chronic or acute disease**		
	Yes	240 (37.3)
	No	404 (62.7)
**Purpose for visiting the OHC**^a^		
	User’s own health issues	377 (58.5)
	As a caregiver	148 (23.0)
	Other purposes	119 (18.5)
Total		644 (100)

^a^OHC: online health community.

**Table 2:** Confirmatory factor analysis of the extended eHealth literacy scale (eHEALS-E). All items are on a scale of 1=completely disagree to 5=completely agree. Only factor weights of absolute value equal or larger than .40 are reported.

Scale items	Fac1^a^	Fac2^a^	Fac3^a^	Fac4^a^	Fac5^a^	Fac6^a^
I know what health resources are available on the Internet.	.61					
I know where to find helpful health resources on the Internet.	.61					
I know how to use the Internet to answer my health questions.	.57					
I have the skills I need to evaluate the health resources I find on the Internet.		.78				
I can tell high-quality from low-quality health resources on the Internet.		.75				
I can easily extract the essential meaning of some health information on the Internet.		.50				
Considering all health information on the Internet, I sometimes find it difficult to select the most relevant for my health.			−.73			
The huge quantity of health information available on the Internet usually confuses me.			−.78			
I do not have any difficulties understanding the terminology used by some online health resources.			.71			
Sometimes, when I am confronted with a health issue, I am not sure where to start searching for information on the Internet.			−.56			
I feel confident using information from the Internet to make successful health decisions.				.66		
Usually, I do not find helpful health information on the Internet.				−.43		
The Internet helps me to make decisions about my health more easily.				.56		
It is important for me to be able to access health-related online information.				.63		
If I do not fully understand health information on the Internet, I try to make sense of it.					−41	
If I do not understand health information on the Internet, I would rather ask somebody for an explanation than to form my own conclusions.					.52	
It is important to me to check health information that I find on the Internet with other resources (such as doctors, books, friends, or relatives).					.46	
I think that most of the health information we find on the Internet can be trusted (R).						.77
I am satisfied with the first health resource on the Internet that can deliver answers to my questions (R).						.64
On the Internet, I prefer reading short and simple health explanations instead of complicated expert clarifications (R).						.63
Cronbach alpha	.75	.81	.80	.75	.52	.70

^a^Fac1 corresponds to the factor *awareness of sources*, Fac2 to *recognizing quality and meaning*, Fac3 to *understanding information*, Fac4 to *perceived efficiency*, Fac5 to *validating information*, and Fac6 to *being smart on the Net*.

An initial item set of 31 items was evaluated for content validity by 3 experts (one in social science methodology, one in health communication, and one in Internet studies), and on this basis, a refined set of 26 items was selected. Exploratory factor analysis did not reveal factors that would fit the four components of eHealth literacy as proposed by Norman and Skinner [13] but unveiled six factors, which, nevertheless, are meaningful and can be coined as awareness of sources, recognizing quality and meaning, understanding information, perceived efficiency, validating information, and being smart on the Net. The name of the last dimension comes from a resemblance to skills that Rheingold [62] identified as crucial in using the Internet in his book *Net Smarts*. This solution was tested with confirmatory factor analysis, which demonstrated an acceptable fit of the proposed model (root mean square error of approximation [RMSEA]=.059, standardized root mean residual [SRMR]=.058, comparative fit index [CFI]=.94). In Table 2, all items per dimension, their factor loadings, and Cronbach alphas per each dimension are listed. Correlations between dimensions (see Table 3) and analysis of average variance extracted (AVE) values (not reported but available from the authors) demonstrated satisfactory discriminant validity, noting somewhat lower discrimination between dimensions of awareness of sources and perceived efficiency. The final scale comprises 20 items, as the communalities of some items were too low on the existing factor solution and had to be excluded from the analysis (including two reverse-coded items from the original eHEALS).

**Table 3:** Correlations between dimensions of the extended eHealth literacy scale (eHEALS-E).

eHEALS-E dimensions	Validating information	Being smart on the Net	Understanding information	Awareness of sources	Perceived efficiency	Recognizing quality
Validating information	1	.34^a^	.07	.16^a^	.10^b^	.11^a^
Being smart on the Net		1	.31^a^	.08	.09^b^	.16^a^
Understanding information			1	.39^a^	.39^a^	.45^a^
Awareness of sources				1	.59^a^	.64^a^
Perceived efficiency					1	.47^a^
Recognizing quality						1

^a^*P*<.01.

^b^.01< *P*<.05.

**Table 4:** Nominal items that measure users’ activities.

Set of nominal items that measure users’ activities	Yes (%)
Did you post any questions for other users on discussion boards within the last 12 months?	23.0
Did you post any questions for health professional moderators on discussion boards within the last 12 months?	37.1
Did you start a new thread on discussion boards within the last 12 months?	24.8
Have you ever posted a message on the discussion boards on MON^a^?	71.0
Did you post answers to other users’ questions within the last 12 months?	23.4
Did you visit social support discussion boards within the last 12 months?	42.3
Did you visit general social discussion boards within the last 12 months?	57.3
Did you visit professional counseling discussion boards within the last 12 months?	82.2

^a^MON: Med.Over.Net.

#### Users' Activities

As there is little evidence for consistent user typology across different OHCs [34], the typology was empirically established based on clustering units by similarities across various participation variables. An extensive set of metrics for participation in OHCs exists [34], of which some are overdetailed or unsuitable for our type of research design. Consequently, we included some of the most common survey-based measures of user activity in an OHC. The first was a set of dichotomous questions that pertain to the participation styles and the type of discussion boards visited (see Table 4 for the wording of the items and the frequencies). The second was a set of ordinal measures that pertain to the length of membership and the frequency of activities in the OHC (see Table 5 for the wording of the items and the frequencies).

### Analyses

In line with previous studies [34], we did not a priori assume the user typology but obtained it empirically with a hierarchical agglomerative clustering algorithm, which iteratively joins the most similar users according to the users’ activity metrics based on Ward’s minimum variance method [63]. More precisely, users were assigned to clusters according to similarities across the 11 users’ activities items above. Cluster membership was stored in a new variable that was used to analyze the research question.

Exploratory factor analysis was conducted to explore the factor structure of the scale to measure eHealth literacy and to determine which items of the scale should be retained. Factors were extracted using principal axis factoring with oblimin rotation, as we did not expect an orthogonal factor solution. The number of factors was selected based on eigenvalues higher than 1. This decision was also supported by inspection of the scree plot. The obtained factor solution was put in a confirmatory factor procedure (using package lavaan in R [64]), which resulted in several statistics that estimate the goodness of fit of the factor model to the study data. As the statistics showed a good fit of the model, no modifications were needed.

To analyze the research question, a one-way analysis of variance (ANOVA) method was used with post hoc pairwise comparison tests to investigate the statistical significance of differences among pairs of user types.

**Table 5:** Ordinal items that measure users’ activities.

Set of ordinal items that measure users’ activities	Values	%
When did you last visit discussion boards on MON^a^?	1-last 7 days	33.4
	2-a week to 1 month ago	28.4
	3-a month to half a year ago	27.8
	4-half a year to a year ago	10.4
How long have you been a user of discussion boards on MON?	1-more than 3 years	56.7
	2-from 1 to 3 years	32.4
	3-less than a year	9.4
	4-less than a month	1.5
How often have you posted messages on message boards on MON within the last 6 months?	1-every day or almost every day	1.2
	2-at least once a week	2.0
	3-at least once a month	4.0
	4-less frequent than once a month or never	92.8

^a^MON: Med.Over.Net.

## Results

### User Typology

Clustering resulted in four meaningful groups of users, who have high within-group similarity and high between-group variance regarding the user activity variables. The emergent typology of the studied OHC overlaps to a great extent with that of the existing studies [34]. If we try not to depart from the nomenclatures of existing studies, then we can name and describe the following four clusters of users with distinct characteristics of their activities in the OHC: active help-seekers, lurkers, core relational users, and low-engaged users (see Table 6).

The largest group of users—active help-seekers—comprises 48.3% (311/644) of the whole sample. This group is characterized by users who regularly visit the OHC, the majority of whom have been members of the OHC for more than 3 years. They mostly participate in help- and advice-seeking behavior by posting messages for health professional moderators, while occasionally also lurking in the online support and socializing sections of the community. The second group comprises typical lurkers and represents 31.8% (205/644) of the sample. They are moderately frequent users of all types of forums on the OHC but never post messages—not for other members and not for health professional moderators. The third group represents 16.9% of the sample (109/644) and includes very active members—core relational members—who are experienced users, with a high frequency of participation in support and social groups and who occasionally also interact with health professional moderators. We can safely claim that these users probably produce most of the user-generated content in the social and support forums. The fourth group, low-engaged users, is the smallest and represents only 3% of the sample (19/644). These are very infrequent, short-term users of the OHC, who have not been in the community for a long time, post questions only in the medical consultation forums, and are not interested in others’ experiences.

The sociodemographic profiles of the clusters of users and their health-related characteristics are presented in Table 7. In this table, row percentages are presented to describe the characteristics of the obtained clusters.

The sociodemographic profiles show several differences among the clusters of users. In the group of active help-seekers, there is the highest percentage of women (72.3%, 225/311) and highly educated (52.4%, 163/311) in comparison with the other three clusters. Conversely, the mean age of the users in this cluster is smaller (mean=38.9 years) in comparison with the other three clusters. In the cluster of low-engaged users, the percentage of men (58%, 11/19) is the highest, and this is the oldest cluster among the four clusters (mean=41.8 years).

**Table 6:** User types on Med.Over.Net (MON).

Cluster	n (%)	Description in terms of typical activities
Active help-seekers	311 (48.3)	Moderately frequent users, long-term members, occasionally post questions, and mostly for health professional moderators; less involved in support and social forums
Lurkers	205 (31.8)	Moderately frequent users, experienced, do not post any sort of messages, and visit all types of forums
Core relational users	109 (16.9)	Frequent users, very frequent posters, experienced members, ask and answer questions, and engaged in discussions in all types of forums
Low-engaged users	19 (3.0)	Infrequent users, rarely open new threads, and post questions only for health professional moderators

**Table 7:** Sociodemographic and health-related characteristics of the user clusters.

Cluster	% of females	% with high education	Mean age in years	% with a long-term chronic or acute disease	% visiting because of own health issues	% visiting as caregivers
Active help-seekers	72.3	52.4	38.9	36.0	60.5	25.2
Lurkers	64.4	36.1	41.4	30.7	53.7	35.1
Core relational users	67.0	45.0	40.4	43.5	62.4	11.0
Low-engaged users	57.9	36.8	41.8	52.6	58.8	17.6

There are also observable differences in the users’ health-related characteristics. Interestingly, the highest percentage of users with a long-term chronic or acute disease is in the low-engaged cluster (53%, 10/19), whereas the smallest percentage is in the lurkers cluster (31%, 63/205). The group with the highest percentage of caregivers is the lurkers cluster (35%, 72/205). In the core-relational group, the percentage of caregivers is the lowest (11%, 12/109), whereas the percentage (62%, 68/109) of those who visit the OHC because of their own health issues is the highest. The remaining 27% (30/109) are using the OHC because of other non-health-related reasons. The design of the research and the limited space in the questionnaire did not allow more detailed analysis of the types of health issues.

### Presence of the eHEALS-E Dimensions

To investigate the levels of eHealth literacy, we first computed the scores for all six eHEALS-E dimensions as obtained with the factor analytical procedures. A series of paired sample *t* tests revealed that awareness of different sources (mean=3.98, SD=0.67) and perceived efficiency (mean=3.94, SD=0.65) are the most developed dimensions of the eHEALS-E, as users score statistically significantly higher in these two dimensions in comparison with all others (*P*<.001). The dimensions of recognizing quality and meaning (mean=3.84, SD=0.80) and validating information (mean=3.80, SD=0.61) are statistically significantly more common (*P*<.001) than being smart on the Net (mean=3.74, SD=0.81) and understanding information (mean=3.11, SD=0.75). Being smart on the Net and understanding information are the least developed dimensions and are statistically significantly less developed (*P*<.001) than all other dimensions. Table 8 reports differences among all dimensions.

### Analysis of Research Question

To analyze the research question, we compared the scores of the eHEALS-E dimensions across the four user groups. We first conducted ANOVA to test differences in the eHEALS-E across groups and then conducted pairwise post hoc tests to determine among which groups the differences are statistically significant (see Table 9).

The user typology is weakly associated with the eHEALS-E dimensions, as the effect sizes are small according to the established guidelines [65]. However, the analysis nevertheless reveals that there are statistically significant differences among the groups of users regarding all six dimensions of eHealth literacy. Post hoc tests reveal that active help-seekers have statistically significantly higher levels of eHealth literacy in comparison with lurkers in four dimensions: understanding information (*P*=.002), awareness of resources (*P*=.002), perceived efficacy (*P*<.001), and being smart on the Net (*P*=.006). Active help-seekers also perform better in all these dimensions in comparison with low-engaged users.

**Table 8:** Means of the extended eHealth literacy scale (eHEALS-E) dimensions and the statistical significance of the mean differences.

Dimension of eHEALS-E^a^ or group	Mean (SD^b^)	Row-VI^c^	Row-UI^d^	Row-AS^e^	Row-PE^f^	Row-RQ^g^
Validating information	3.80 (0.61)					
Understanding information	3.11 (0.75)	0.69^g^				
Awareness of sources	3.98 (0.67)	−0.18^h^	−0.87^h^			
Perceived efficiency	3.94 (0.65)	−0.14^h^	−0.83^h^	0.04		
Recognizing quality	3.84 (0.80)	−0.04	−0.73^h^	0.14	0. 10^i^	
Being smart on the Net	3.74 (0.78)	−0.06	0.63^h^	−0.24^h^	−0. 20^h^	−0. 10^i^

^a^eHEALS-E: extended eHealth literacy scale.

^b^SD: standard deviation.

^c^VI: validating information.

^d^UI: understanding information.

^e^AS: awareness of sources.

^f^PE: perceived efficiency.

^g^RQ: recognizing quality.

^h^*P*<.01.

^i^.01< *P*<.05.

**Table 9:** Comparison of user types across dimensions of the extended eHealth literacy scale (eHEALS-E).

Dimension of extended eHealth literacy scale or group	Active help-seekers (N=311)	Lurker (N=205)	Core relational users (N=109)	Low-engaged (N=19)	Whole sample (N=644)	Significance of *F*-statistics	Effect size (eta-squared)
Validating information	3.84^a,b^	3.83^c^	3.64^a,c^	3.57^b^	3.80	.01	0.02
Understanding information	3.20^a,b^	2.97^a,c^	3.16^c,d^	2.75^b,d^	3.11	<.001	0.02
Awareness of sources	4.06^a,b^	3.87^a,c^	4.02^c,d^	3.69^b,d^	3.98	<.001	0.03
Perceived efficiency	4.02^a,b^	3.81^a,c^	3.98^c,d^	3.58^b,d^	3.94	<.001	0.03
Recognizing quality	3.88^a^	3.72^a,b^	3.98^b^	3.78	3.84	.05	0.01
Being smart on the Net	3.84^a,b^	3.64^a^	3.67^b^	3.59	3.74	.02	0.02

^a^Group has statistically different mean value (*P*<.05) of the corresponding row dimension of eHEALS-E in comparison to the mean value of the other group with the same superscript.

^b^Group has statistically different mean value (*P*<.05) of the corresponding row dimension of eHEALS-E in comparison to the mean value of the other group with the same superscript.

^c^Group has statistically different mean value (*P*<.05) of the corresponding row dimension of eHEALS-E in comparison to the mean value of the other group with the same superscript.

^d^Group has statistically different mean value (*P*<.05) of the corresponding row dimension of eHEALS-E in comparison to the mean value of the other group with the same superscript.

In comparison with lurkers, core relational users have higher levels of eHealth literacy in the dimensions of understanding information (*P*=.04), perceived efficiency (*P*=.03), and recognizing quality (*P*=.01). In contrast, core relational users perform worse than lurkers in the dimension of validating information (*P*=.01). Core relational users also score worse in this dimension of eHealth literacy in comparison with active help-seekers (*P*=.005). More importantly, core relational users have significantly lower scores for the being smart on the Net dimension in comparison with active help-seekers (*P*=.05).

Low-engaged users perform the worst in terms of eHealth literacy, as they score significantly lower in the dimension of understanding information in comparison with the other three groups. Low-engaged users also score significantly lower than active help-seekers in the dimensions of awareness of sources (*P*=.02) and perceived efficacy (*P*=.004). Other differences are also notable, but they are not statistically significant because of the small size of this group.

## Discussion

### Variable Presence of Different Dimensions of eHealth Literacy

The main aim of this research was to investigate the levels of eHealth literacy that various types of users of OHCs possess. To investigate this question, we first revised and extended the existing measurement instrument for eHealth literacy in light of numerous criticisms of the eHEALS [5,54]. With the inclusion of additional items that tap into various essential components of the eHealth literacy concept, the data surprisingly unveiled a set of six distinct yet meaningful dimensions of the eHEALS-E. These dimensions are developed to different extents in the sample of OHC users. Awareness of different health-related sources on the Internet and self-assessed efficiency in search and use of health-related information are the most common dimensions of eHealth literacy across all groups of OHC users, whereas the other dimensions are less common. Interestingly, skills for using the Web smartly and skills for understanding the information are the least developed. The latter is quite expected, as understanding medical information demands a high level of health literacy coupled with professional knowledge [12].

Whereas previous research demonstrated one or at the most two dimensions of eHealth literacy, the eHEALS-E reveals six dimensions. Although we added items to more thoroughly represent components of accessing, understanding, appraising, and applying health-related online information, these items combine in a different manner than was theoretically assumed [12]. For example, the original access dimension seems to resonate in the dimension awareness of Internet sources, whereas perceived efficiency seems to integrate elements of efficiency in the accessing and applying the information dimension. The dimension understanding is supplemented with that of validating information. The dimension that we coined, being smart on the Net, seems to be an important and distinct part of eHealth literacy, which has not been satisfactorily considered by the existing measures.

This point is also emphasized, at least indirectly, by the authors of the electronic health literacy scale (e-HLS; [5]), a recently introduced eHealth literacy scale, which unfortunately could not be considered in this study, as we had already collected the data. Whereas the eHEALS-E builds on the theoretical underpinnings of the eHEALS, the e-HLS builds on a somewhat broader set of studies and is represented by items that measure different activities that users undertake when browsing online resources (checking credentials, last update, etc), trust in online information, and communicating about information obtained with health providers [5]. Although the items in the e-HLS are rather different, the eHEALS-E similarly tries to incorporate skills assumed by the e-HLS by considering the perspectives of cross-validating information with colleagues and health providers, the ability to discern reliable sources from unreliable ones, and critical appraisal of online information. The main difference is that items in the eHEALS-E measure not only activities but also self-assessed skills (similar to the eHEALS) and attitudes. The latter, in our opinion, are more subtle at revealing skills and practices (or the lack of them) that pertain to the most critical issues of appraising online information. In this way, we managed to discern the presence of the so-called *bad literacy* phenomenon [39], which is the phrase we coined and computed in our study in its reverse meaning (being smart on the Net) for methodological reasons to compare the dimensions. High correlations between the items that compose this dimension (between “I’m happy with the first search result that I get, when I search for health information” and “I think that most of the health information that we can find on the Internet can be trusted,” and “When I get useful information on the Net, I’m not interested who is its author”) clearly demonstrate practices that show the absence of an important dimension of eHealth literacy—the one that deals with the awareness of misinformation and biases in search engines and popular social media [58-60]. Moreover, this dimension of eHealth literacy combines a lack of interest in verifying authorship, coupled with a naïve trust in the objective gatekeeping function of Internet search engines. In comparison with the e-HLS, trust in this study appears here as a component that diminishes the critical appraisal of online health information and thus, diminishes eHealth literacy. In any case, we believe that complementing the theoretical and operational perspectives of the eHEALS-E and the e-HLS would lead to higher quality of eHealth literacy measurements.

### The Users Who Participate the Most Are Not the Most Literate

The analysis of the newly introduced eHEALS-E among users of OHCs reveals several noteworthy findings. First, we demonstrated, theoretically and empirically, that it is important to distinguish between different types of users in investigating eHealth literacy. Active help-seekers, a common user type in other studies of OHCs [34], are, interestingly, the most literate users in almost all dimensions of eHealth literacy in comparison with other user types. These users have the skills to navigate smartly around the Internet, recognize information biases, and validate the information obtained through their colleagues and professional sources. However, these users scored a bit lower on the dimension of recognizing quality, revealing limited medical knowledge for directly recognizing the quality and essence of health-related information and its implications for their own decision making and actions. The likely consequence of this is that the group’s use of OHCs is characterized by tailor-made queries in professional consultation forums, where they can find clarifications and illustrations of professional knowledge [31]. As the group of active help- seekers is the most literate in all other dimensions of the eHEALS-E, this group likely has an important positive cumulative effect on the whole OHC. The many questions that these users post to health professional moderators enable lurkers, who are less literate, to browse intelligibly through the questions and answers. Moreover, results show that the group of active help-seekers is (comparably) female dominant and highly educated. The majority of users within this cluster use the OHC because of their own health issues, yet more than a quarter of the cluster is composed of caregivers. It is important to notice that active help-seeker have relatively high eHEALS-E scores, which likely helps them to provide adequate care to their close ones.

As a group of users, lurkers (who never participate by asking questions or sharing their knowledge but only browse discussions) in general have lower eHEALS-E scores in comparison with the other groups of active participants. As posters and lurkers can reach similar levels of psychological empowerment [19,20], it becomes important that OHCs enjoy high enough levels of valid information. If users were able to build awareness that they can cope with the given health issue and have control over it [18,20] based on low eHealth literacy and the questionable validity of information in OHCs, this would lead to conflicts in relationships with physicians [39] and worse health outcomes in general [66]. Moreover, we found that the highest percentage of caregivers was among the lurkers. Thus, although lurkers do not have a direct impact on the OHC, they have an impact on people to whom they offer health care. Since lurkers score relatively low in the majority of the eHEALS-E dimensions, there is a danger that this group’s interpretation of the information they obtain might not result in the most efficient care for their family members or friends.

On the basis of user activity metrics, we identified another very small group of users that seems to be the most problematic in terms of eHealth literacy. This group, which we, similarly as in some other studies [67], identified as low-engaged users, has the lowest percentage of highly educated users, lowest percentage of females, is the oldest in comparison with other groups, and has the highest percentage of users with long-term chronic or acute illness. On the one hand, members of this group figure the lowest in terms of validating, understanding, and being smart users of the Internet for health-related information, but on the other hand, they are self-confident and trust in their abilities to recognize quality information and grasp the essence from Internet-obtained health information. As items of the latter dimension mostly rely on a self-reported belief in one’s own competence, we can note here a divergence between how people assess their skills and the true nature of their skills. Low-engaged users might be convinced that they get from the Internet the essential health information that they need, but they don’t care much about the validity of that information. This issue becomes more crucial with the most active group of users.

Core relational users represent users who are the most active in the OHC, and their contributions likely have the largest impact on the OHC. This user type probably confounds the more detailed subtypes found in other studies [34]. In comparison with other groups, this group is female dominant and has the smallest percentage of caregivers. The level of eHealth literacy of core relational users is similar to that of active help-seekers, but core relational users perform significantly worse in the dimensions validating information and being smart on the Net. Even in comparison with lurkers, core relational users’ *bad literacy* is significantly higher. In other words, whereas core relational users are very confident about their ability to grasp essential health information efficiently by browsing the Internet, they are mostly unaware of the dangers of biases and misguiding websites, which unarguably exist [58,60]. This finding should be a cause for some concern. Participation by core relational users is strongly motivated by identification with the community. Interestingly, this identification is based mostly on benevolence trust, not on integrity trust [36]. In other words, for a user of an OHC to belong to a community, it is not as important that other users are truthful, consistent, and honest in their messages but that they show concern for them. Consequently, participating users with a strong sense of belonging are not concerned as much about the credibility of their messages but that their behaviors are aligned with the group norms and beliefs. In the context of the finding that users perceive peer advice in OHCs as very credible [30], this opens up affordances of OHCs for the spread and domination of misinformation, especially when group norms and beliefs support unprofessional or unhealthy practices.

Current research does not provide much empirical evidence for the suggestion that such affordances are abused by a minority of motivated individuals to control the discourse in the community [46,61], but it is worrying that discussions in OHCs are very rarely equipped with references to external professional websites [46] and that users reject advice from credible websites when they are not in accordance with their beliefs and lifestyles [68]. Thus, there is a great concern that core relational users could be misguided by pseudoscientific research, as they do not have the expertise to judge the reliability and credibility of the information [46]. In turn, this can have real consequences, as the information these users publish becomes available to other OHC users who can use it to build knowledge about a particular issue and even apply the information to manage their own condition. This study does not provide evidence for existence of such process in the studied OHC. However, we claim that core relational users might present a certain risk for the production of credible OHC knowledge because of their relatively lower developed eHealth literacy in the dimension being smart on the Net. Patients can develop feelings of empowerment and control over their health decisions through social support in OHCs [16,18], but as ideological similarity might be more important than the credibility of the information in judging the quality of peer support [32], such empowerment does not necessarily lead to better health outcomes. On the contrary, when information published by people with low eHealth literacy is validated by other people with low eHealth literacy, users of OHCs can get empowered on invalid bases, which could lead to serious negative consequences [69]. Moreover, as such knowledge becomes prevalent, it provides a template for other members to follow [31], thus resulting in problematic collective behaviors such as in the case of the antivaccination movement [56] or pro-anorexia forums [41].

### Practical Implications

The discussion above provokes practical considerations for OHC managers. Existing research on misinformation in OHCs suggests interventions either at the level of procedures of artificial intelligence in selecting and detecting problematic information and/or regarding the importance of the role of health professional moderators in OHCs. This research adds another practical implication for OHC managers. Occasional assessments of eHealth literacy among core relational users could help identify potential risks for the quality of published information, especially if critical elements of eHealth literacy start decreasing. This way, site managers could intervene before actual misinformation or noncredible information gets published and spreads among the community. The core relational users’ lower values for the dimension being smart on the Net clearly indicate that the site managers of the studied OHC should be more attentive toward this segment and plan suitable interventions. These should be created with great care, as core relational users and their everyday conversations that satisfy different motives are important for successful sustainable online communities [47]. If such conversations are intermeshed with the sharing of health information of questionable validity, this becomes a problem for the community. One mechanism could be to engage health professional moderators, who mostly participate in consultation forums, to enter discussions where core relational users with low eHEALS-E scores dominate the discussion. Another preventive measure might be to provide mechanisms that discourage the closure of conversational space. In other words, users should be stimulated to build bridging social capital, encounter different perspectives on an issue, and not be limited to relatively isolated islands of known and similar persons. This, for example, could be stimulated by awarding participation in different areas of the community.

Furthermore, users should be encouraged by positive awards to support their statements in the form of links to credible websites, as this increases the quality of conversation, which further affects the nature of the impact on health outcomes [70].

In light of the findings above, we find the mechanisms that bring together users on the basis of matchmaking and similar beliefs problematic [26,28], as they might attenuate the bubble effect. It is true that OHCs and online communities in general (most notably Wikipedia) have a sort of self-correcting mechanism by which inaccurate and invalid information is corrected by peers. However, the success of such a mechanism is based on the assumption that contributors have high eHealth literacy. When this assumption is not justified, a community could quickly build on false knowledge and nurture problematic ideas or practices.

In addition, although the awareness of the need for the strong presence of health professional moderators is growing as they filter information, provide links to external websites, and so on [31,43], their role could be further expanded. As suggested above, they should be encouraged to participate or at least lurk in more socializing-oriented parts of the forum. In this way, health professionals could not only discover problematic contents but also inform their professional practice and that of their colleagues about the misinformation and ambiguous discourses regarding symptoms, treatments, or remedies.

### Limitations

The research design used in this study faces several methodological issues that limit the generalizability of the results. For one, this study focused on a single OHC in a specific national context. On the positive side, this OHC is large and encompasses different types of forums, thus resembling OHCs that are more internationally known (such as PatientsLikeMe, WebMD, and MedHelp). We should add that Slovenia is one of the most typical EU countries regarding usage of information and communication technologies. According to many of Eurostat’s informa­tion society indicators, the country is close to the median position among all EU countries [71]. In addition, although we used simple random sampling on the basis of a list of all OHC users, there are certain limitations. The total response rate (4.2%) is not uncommon with this type of research designs [72] but nevertheless is small. We can assume from other studies [73,74] that users who more frequently use and post in the OHC are also more likely to provide responses on the survey. Consequently, the proportions of user types are not representative of the whole OHC. It is likely that the sizes of the lurkers and low-engaged users groups are underestimated, whereas the sizes of the core relational users and active help-seekers groups are probably overestimated. One implication of this limitation is that under a different data collection procedure, which would allow better representation of low-engaged users, the clustering algorithm might reveal a higher variability in user types among low- and nonactive users. For instance, we might get a so-called butterfly user type, which was identified in several other studies [34] as a group of users who visit OHCs frequently but spend short amounts of time per session and jump from one discussion board to another. In addition, among lurkers we might be able to distinguish between short-term users, searching for usable information and long-term users who eventually become active users. Another methodological implication of this limitation is that future studies should focus on the use of various methods for recruiting less- or nonactive users. However, we should be aware that though such methods (eg, log analysis or automated data analysis) might be useful for detecting more complex types of users, they need to be combined with methods that are more informative about the cognitive, emotional, and other psychosocial process in OHCs to be able to measure such tacit phenomena as eHealth literacy.

Furthermore, the data refer only to a specific population of OHC users and do not allow any comparison with the general population. Consequently, we do not know whether the average eHealth literacy level of OHC users is similar to the eHealth literacy of the general population. We can compare only the scores of individual items that are the same as those used in studies in other national contexts [1-3,6] and realize that the scores are fairly similar. It does not seem that the OHC users, on average, would be very different from the general population. However, this is more of a speculation than a scientifically validated statement. This issue is connected to the testing of the eHEALS-E scale. Although tests on the specific sample proved that the revised and extended scale has a meaningful structure, the eHEALS-E scale needs to be retested on a different population and in different national contexts. Moreover, to get a stronger confirmation of validity, criterion validity should be assessed by associating the scale with the outcome measures.

### Conclusions

In this study, we identified different types of OHC users who perform differently regarding eHealth literacy and affect the production of knowledge in this OHC. The proposed extended version of the eHEALS scale, which in our opinion more validly taps various dimensions of this complex construct, allowed us to gain a more nuanced insight into the differences among various types of users. We specifically exposed the core relational users who represent a group of users that produce the most content in OHCs and at the same time show less-developed skills for cross-validating the information obtained and navigating successfully through the perils of the online world. OHC site managers should better monitor these users’ activities to avoid the spread of misinformation and unhealthy practices. However, the value of OHCs should not be rejected despite some rather worrisome findings of this research about the credibility of the information shared in OHCs. Existing research demonstrates many benefits of participation in the OHCs for users and patients in dealing with health issues. However, further research is needed to focus on the early discovery of potential problems in OHCs to eliminate them and to prevent a loss of credibility of the information shared in OHCs. We believe that investigating different dimensions of the eHEALS-E across different types of users can provide important help in this process by discovering segments of users who publish information based on low eHealth literacy, and as such present a risk of growing into a dominant social force that could change the nature of the OHC in an unexpected and harmful way.

## Appendix

Multimedia Appendix 1 Checklist for Reporting Results of Internet E-Surveys.

## Abbreviations

ANOVA: analysis of variance
AVE: average variance extracted
CFI: comparative fit index
CHERRIES: Checklist for Reporting Results of Internet E-Surveys
eHealth: electronic health
e-HLS: electronic health literacy scale
eHEALS: eHealth literacy scale
eHEALS-E: extended eHealth literacy scale
EU: European Union
OHC: online health community
MON: Med.Over.Net
RMSEA: root-mean-square error of approximation
SD: standard deviation
SRMR: standardized root-mean-square residual
WMA: World Medical Association
