# Supplementary material for: Ill Literates or Illiterates? Investigating the eHealth Literacy of Users of Online Health Communities
Source: J Med Internet Res. 2017 Oct 4;19(10):e331. doi: 10.2196/jmir.7372 (PMC5647457; doi:10.2196/jmir.7372)
Supplement: Multimedia Appendix 1 [file jmir_v19i10e331_app1.pdf]

Multimedia Appendix 1

Checklist for Reporting Results of Internet E-Surveys (CHERRIES) – eHEALS-E

Item Category	Checklist Item	Explanation
**Design**	Target population	Registered users of Slovenian online health community Med.Over.net (MON). List-based sampling frame.
	Survey topics	Use of various internet services to obtain health-related information, e-health literacy, activities in MON; user's experience and satisfaction with MON.
**IRB approval and Informed consent**	IRB approval	As per the code of ethics for researchers at the University of Ljubljana, no institutional ethics approval was needed for this retrospective type of study. Research was conducted in line with the WMA Declaration of Helsinki on ethical principles for medical research involving human subjects.
	Informed consent	After clicking the link for the Web survey in the email, potential respondents were taken to an informed consent Web page with information about the purpose of research and the length of the survey, an assurance that the data would be dealt with in accordance with national and EU laws, information on who the investigator was, a contact information and a statement that they were under no obligation to participate, and that the aggregated results may be published.
	Data protection	MON is a reputable Web service that treats all personal information (emails) in accordance with national and EU laws and protects data with standard security procedures, which include the deidentification of locally held data files, physical protection of hardware, and strong password protection. The authors of this study had no access to the emails of respondents and received an anonymized dataset containing no identifiable personal information.
**Development and testing**	Development and testing	The survey was developed in collaboration with the providers of MON as a part of their annual survey on user experiences and satisfaction with the OHC. Survey development was supported by

		one survey design specialist at the University of Ljubljana. The survey was pilot tested by five postgraduate students in Social Informatics trained in survey design and item development.
**Recruitment process and description of the sample having access to the questionnaire**	Open survey versus closed survey	The survey was opened to participants who registered on the MON with a valid e-mail address.
	Contact mode	The OHC provider invited potential respondents to participate in the Web survey via its email newsletter service.
	Advertisting the survey	Invitation e-mail advertised the survey as an annual survey on user experiences and satisfaction with the OHC with information on additional module, introduced as a scientific inquery of the role of OHC in society. The e-mail invitation was designed in line with recent guidelines for desiging e-mail invitations for web surveys on online community users.
**Survey administration**	Web/email	The survey was administered by OHC providers through e-mail newsletter service.
	Context	The e-mail invitation contained solely information on the survey.
	Mandatory/voluntary	Participation in the survey was voluntary.
	Incentives	No incentives were used.
	Time/date	The survey was administered and available from June 1st to June 30th, 2016.
	Randomization of items of questionnaires	Some sets of items were randomized within an items-table, while variables were not randomized in order to retain a logical structure of the whole questionnaire.
	Adaptive questioning	Where relevant, conditioning and routing was used.

	Number of items	In general the survey was structured in one measurement instrument per page format. Except where measurement instrument contained more than 10 items, the table was split in additional pages.The maximum number of items per respondent was 181, while the total number of items was larger as several measurement experiments were conducted on instruments, which were not used for this study and are thus not reported. The respondents took in average 19 minutes to complete the survey.
	Questionnaires submitted with an atypical timestamp	Not relevant.
	Number of screens (pages)	The total number of pages a participant could see was 39, but due to skip patterns the total number of pages was lower.
	Completenss check	A completeness check was conducted after the questionnaire was submitted during the analysis phase. The questions did not require a response in order to advance to subsequent questions, except in case of routing questions.
	Review step	Due to the length of the survey, participants were not required to review their responses at survey completion. A “back” button was provided if participants wished to edit previous answers.
**Response rates**	Unique site visitor	The system in which web survey was administered (http://english.1ka.si/) uses both cookie and IP based mechanisms to determine unique site visitor.
	View rate	It is not possible to calculate.
	Participation rate	10.71% (1607/15000)
	Completition rate	41.88% (673/1607)
**Multiple entries**	Cookies used	Cookies were used and stored for a duration of maximum 1 month. Respondents were informed about cookies in line with the EU cookie policy.
	IP check	IP addresses were not collected from participants, but the survey system collects them and prevents multiple entries from the same IPs.

	Log file analysis	No log file analysis were performed.
**Analysis**	Handling of incomplete quesitonnaiers	Units which provided item nonresponse on the concepts that were used in the analysis were exlcuded from the analysis. If the remaning units had some nonresponse on socio-demographical variables (which were placed at the end of the questionnaire) missing value imputation was performed on socio-demographic variables.
	Statistical correction	No statistical correction procedures or weightings were used in the analysis.
